# Appearance of fast astrocytic component in voltage-sensitive dye imaging of neural activity

**DOI:** 10.1186/s13041-015-0127-9

**Published:** 2015-06-05

**Authors:** Ildikó Pál, Julianna Kardos, Árpád Dobolyi, László Héja

**Affiliations:** Group of Functional Pharmacology, Institute of Cognitive Neuroscience and Psychology, Research Centre for Natural Sciences, Hungarian Academy of Sciences, Magyar tudósok körútja 2, H-1117 Budapest, Hungary; MTA-ELTE-NAP B Laboratory of Molecular and Systems Neurobiology, H-1117 Budapest, Hungary; Department of Anatomy, Human Brain Tissue Bank, Semmelweis University, H-1450 Budapest, Hungary

**Keywords:** Voltage-sensitive dye, Intrinsic optical signal, Astrocyte, Excitatory amino-acid transporter type 2, Hippocampus, Field potential, Photodiode-array

## Abstract

**Background:**

Voltage-sensitive dye (VSD) imaging and intrinsic optical signals (IOS) are widely used methods for monitoring spatiotemporal neural activity in extensive networks. In spite of that, identification of their major cellular and molecular components has not been concluded so far.

**Results:**

We addressed these issues by imaging spatiotemporal spreading of IOS and VSD transients initiated by Schaffer collateral stimulation in rat hippocampal slices with temporal resolution comparable to standard field potential recordings using a 464-element photodiode array. By exploring the potential neuronal and astroglial molecular players in VSD and IOS generation, we identified multiple astrocytic mechanisms that significantly contribute to the VSD signal, in addition to the expected neuronal targets. Glutamate clearance through the astroglial glutamate transporter EAAT2 has been shown to be a significant player in VSD generation within a very short (<5 ms) time-scale, indicating that astrocytes do contribute to the development of spatiotemporal VSD transients previously thought to be essentially neuronal. In addition, non-specific anion channels, astroglial K^+^ clearance through K_ir4.1_ channel and astroglial Na^+^/K^+^ ATPase also contribute to IOS and VSD transients.

**Conclusion:**

VSD imaging cannot be considered as a spatially extended field potential measurement with predominantly neuronal origin, instead it also reflects a fast communication between neurons and astrocytes.

## Background

Understanding brain (dys)functions necessitates valid representations of spatiotemporal dynamics of neural activity. For this purpose, two major approaches are used, the electrophysiology based multielectrode arrays (MEA) [1] and the optical signal based methods [2]. The MEA-based activity mapping takes advantage of the well-characterized electrophysiological signal with exceptionally high temporal resolution. However, the method also suffers from several important drawbacks, including the limited spatial resolution and severe invasiveness. Spatiotemporal optical imaging approaches offer a solution to overcome these limitations. However, despite the considerable efforts to uncover the cellular and molecular processes underlying the genesis and spreading of optical signals [3–6], important details of these processes and especially the role of glial cells and neuro-glia interactions still remained obscure. We addressed the identification of neuronal and glial target proteins underlying optical imaging of neural activity by comparing two widely used [7–12] optical methods, the intrinsic optical signal (IOS) and the voltage-sensitive dye (VSD) imaging.

The label-free IOS [13], enabling non-invasive mapping of patients’ neuronal activity under intra-operative conditions [14, 15], generally considered to measure light scattering changes [8] due to activity-dependent cell swelling [5, 8, 16]. Recently, however, several other physiological processes, including astroglial glutamate uptake and neuro-glia coupling has also been identified as significant contributors to the generation of intrinsic optical signal [17–22].

VSDs bind to the extracellular part of the cell membrane and transforms electrical activity to optical signal [4]. In contrast to IOS, the VSD transients operate on time scale similar to field potential (FP) recording, however, significant differences in the signal length of locally recorded FP and VSD transients have also been observed [23–25]. VSD imaging is typically considered as the optical counterpart of FP measurements and is used for expanding the one-dimensional FP when the multi-electrode arrays cannot be applied due to their invasiveness or low spatial resolution [26–28].

To determine how neuronal and astroglial activity is coupled to spatiotemporal optical signals we measured the regional distribution and spreading pattern of IOS and VSD transients following brief Schaffer collateral stimulation in rat hippocampal slices and compared the two spatiotemporal signals to each other and also to the FP measured locally in the CA1. By pharmacological inhibition of several possible neuronal and astroglial molecular targets (Glu receptors, major astroglial Glu transporter, neuronal K^+^/Cl^−^ cotransporter KCC2, Na^+^/K^+^/Cl^−^ cotransporter NKCC1, non-specific Cl^−^ channels, K_ir4.1_ potassium channel and Na^+^/K^+^ ATPase) we assessed their role in IOS and VSD signal generation.

## Results

### Spatiotemporal pattern of stimulation evoked VSD and IOS transients in the rat hippocampal slice

**Fig. 1:**
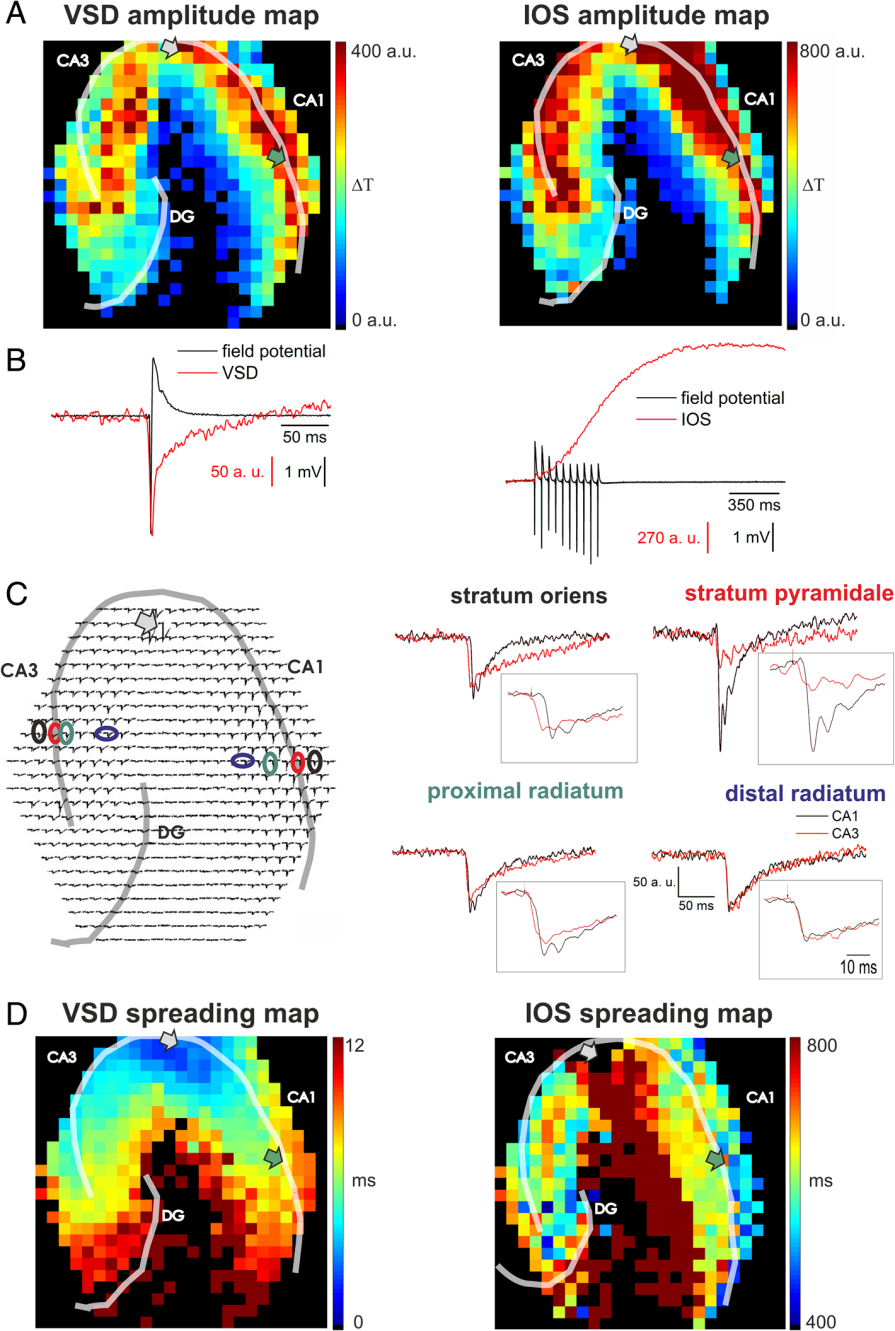
Spatial characteristics of VSD and IOS transients. **a**: 2D representation of the VSD (left) and IOS (right) amplitude distribution in the hippocampal slice. Optical signals were recorded from the same NK3630 stained slice. **b**: Time course of the field potential responses and the VSD (left) and the IOS (right) transients on the diode at the field potential recording site evoked by a single stimulus (VSD) or a stimulus train (10 stimuli, 20 Hz, IOS). Signals were recorded from the two different slices. IOS was recorded from an unstained slice while VSD was recorded from a stained slice. **c**: (*Left*) Spatial overview of the VSD transient pattern on the 464-element photodiode array. Transparent lines indicate the pyramidal cell layers (CA1 and CA3) and the granular cell layer of the DG. The position of the stimulating electrode is marked by an arrow. (*Right*) Comparison of the profile of VSD traces along the somato-dendritic axis of CA1 (black lines) and CA3 (red lines) regions. Inserts show the first 40 ms of the traces following stimulation onset. **d**: 2D representation of the VSD and IOS spreading patterns. Spreading is represented by the time required for the optical signal to reach 50 % of its amplitude following the stimulus onset. Transparent lines indicate the pyramidal cell layers (CA1 and CA3) and the granular cell layer of the DG. The positions of the stimulating and recording electrodes are marked by gray and green arrows, respectively

To assess the molecular determinants of IOS and VSD signals we stimulated the Schaffer collateral in 400 μm thick rat hippocampal slices and measured the resulting changes in the label-free intrinsic optical signal (IOS) and in the absorbance of the NK3630 voltage-sensitive dye (VSD) in the whole hippocampus with a high-frequency sampling rate (~1.6 kHz) imaging device, a 464-element photodiode array (PDA) detector. The high sampling frequency of the PDA enables the temporal alignment of the optical signals with the simultaneously measured field potential (FP). Fig. 1b shows the relation between the time scales of the optical signals and the FP. IOS is approximately 12 times longer than the FP, while the length of the VSD transient is similar to the FP time scale.

To determine whether optical signals correlate to the electrophysiological signal, the amplitude of the population spike (PS) measured in the CA1 *str. pyramidale* was compared to the summa amplitude of each optical signal in the whole CA1. The amplitude of the population spike measures the synchronous firing of the neighboring neurons [29]. In addition, to further strengthen the parity of the electrophysiological and optical signals, PS amplitude was also compared to the optical signal amplitudes measured on the single diode at the FP recording site. Furthermore, summa amplitudes of the IOS and VSD signals in the CA1, CA3 and *dentate gyrus* (DG) regions of the hippocampus were also compared to each other to evaluate regional differences in the generation of the two optical signals.

Both IOS and VSD amplitudes measured in the close vicinity of the recording electrode in the CA1 pyramidal layer were found to be linearly correlating with the PS amplitude (*R*^*2*^ *= 0.98*), suggesting that IOS and VSD linearly follow neuronal activity. Spatial distribution patterns of VSD and IOS showed highest amplitudes in the CA1 *str. pyramidale* and proximal *str. radiatum* (Fig. 1a), corresponding to the innervated area of the stimulated Schaffer collaterals. Both signals were also detected in the CA3 pyramidal layer most probably due to antidromic stimulation [30] and the activation of the CA3 to CA3 associational projections [31]. In 72/100 slices the activated area also included the DG showing the activation of mossy fibers.

The amplitude distributions in the CA1 were similar for the VSD and IOS signals, but they were found to be different in the CA3 region. In the CA3 region IOS showed the highest amplitude transients in the pyramidal layer and in the proximal radiatum, while the highest VSD amplitude was observed in the distal radiatum. This phenomenon can arise from the different way of stimulation (orthodromic vs. antidromic) or the different mechanisms underlying the two optical signals.

The dynamics of both optical signals were also significantly different in the CA3 and CA1. In the antidromically stimulated CA3, IOS transients in the *str. radiatum* were found to be smaller than in the pyramidal layer, while in the orthodromically activated CA1, IOS traces in the *str. radiatum* overshot those recorded in the *str. pyramidale* [5]. Contrary, the VSD transients in the CA3 *str. radiatum* were higher than in the pyramidal layer, while in the CA1 transients in the *str. radiatum* were smaller than in the pyramidal layer (Fig. 1a). The subregional activation pattern of the VSD was also different in the CA1 and CA3 as traces in the *str. oriens* of the CA1 rose significantly later than the traces in the *str. radiatum,* opposite to the pattern obtained in the CA3 (Fig. 1c insert). Despite of the differences between the time scales of the two optical signals, both signals can be applied to discriminate between orthodromic (CA1) and antidromic (CA3) activation patterns.

Shapes of the VSD signals in the *str. pyramidale* and *str. oriens* of the CA1 were different from those of the CA3 but in the *str. radiatum* they were very similar in the CA1 and CA3 (Fig. 1c).

The high-frequency sampling rate of the PDA device allowed us to temporally dissect IOS and VSD generation processes (Fig. 1d). As it was previously shown [5], IOS appears first in the CA1 *str. pyramidale* at Schaffer collateral end points than in the *str. radiatum* and other parts of the *str. pyramidale* of the CA1 and CA3. Subsequently, it spreads along the pyramidal layer in the *str. radiatum* towards the stimulating electrode and the subiculum [5].

There are several possible explanation for the observed temporal appearance of IOS. Schaffer collaterals surround the pyramidal layer from both sides (*str. radiatum* and *str. oriens*) [32], therefore the somatic layer might have a higher concentration of neurotransmitters to induce IOS. Another explanation is that IOS reaches detection limit earlier in the somatic region of pyramidal cells than in the dendritic region. Since the extracellular volume is the smallest in the pyramidal layer in the CA1 [33], small changes in cell volume in this layer might lead to drastic changes in light scattering. Alternatively, neuronal swelling might arise faster than glial swelling. It is still not clear how neurons swell under physiological conditions [34]. There is evidence that the swelling of the axons is a fast process, it occurs on a similar time scale to action potential generation (1–10 ms) [35–37] so swelling of the axon initial segment may be observed earlier. This is in agreement with the relatively early IOS response in the *str. oriens*. Since cortical neurons lack functional water channels, Andrew and coworkers supposed that neurons swell by opening non-aquaporin channels to water [38], like potassium channels that are permeable to water [39].

The VSD signal had completely different temporal appearance and spreading pattern. Following the stimulation, VSD signal appeared first near the stimulating electrode than spread along the pyramidal layer towards the subiculum or the DG, parallel in the CA1 and the CA3 (Fig. 1d). It is to note that the two signals are on a different time scale, VSD traverses the hippocampal slice in ~12 ms while IOS needs more than a second to fully propagate. Therefore the spreading pattern of IOS does not follow the sequence of neural depolarization indicated by the VSD signal. The estimated spreading velocity of the VSD signal is 300 ± 80 μm/ms, which is three orders of magnitude faster than that of the IOS (0.23 ± 0.05 μm/ms) and is in the range of the spreading velocity of the field potential signal (240 ± 12 μm/ms), showing that VSD imaging can reliably follow neuronal activity.

### Neuronal activation is required for IOS and VSD

To examine whether VSD signal is evoked by synaptic activation following the Schaffer collateral stimulation we applied the voltage-gated sodium channel inhibitor tetrodotoxin (TTX, 5 μM). TTX completely eliminated the PS (0 ± 1 % of control, N = 5) and the VSD signals (1 ± 0.4 % of control, N = 5), demonstrating that the passive voltage propagation in the tissue is insufficient to evoke VSD. Therefore VSD signal is strictly initiated by neuronal activation.

Enhancement of neuronal activity by applying the GABA_A_ receptor antagonist picrotoxin (100 μM) increased both PS (357 ± 26 % and 312 ± 20 % of control, N = 5 and N = 8 for IOS and VSD measurements, respectively) and optical signal amplitudes in all the examined regions to 135–200 %. The picrotoxin-induced increase of the optical signal amplitudes in the CA1 was less pronounced than that of the PS amplitude (PS_IOS_: 468 ± 46 %, PS_VSD_: 592 ± 71 %, IOS in CA1: 135 ± 17 %, VSD in CA1: 182 ± 12 % of control). Increase of IOS amplitude was also significantly smaller than that of VSD in the CA3 region (IOS in CA3: 137 ± 11 %, VSD in CA3: 168 ± 10 % of control). Interestingly, the changes in the VSD signal showed no regional differences, while IOS increase was significantly more pronounced in the DG than in the CA1 and the CA3 regions. Both optical signals were also increased at the FP recording site (IOS: 192 ± 49 %, VSD: 158 ± 10 % of control).

### Blockade of astrocytic Glu uptake distinguishes among FP, IOS and VSD measures of neural activity

**Fig. 2:**
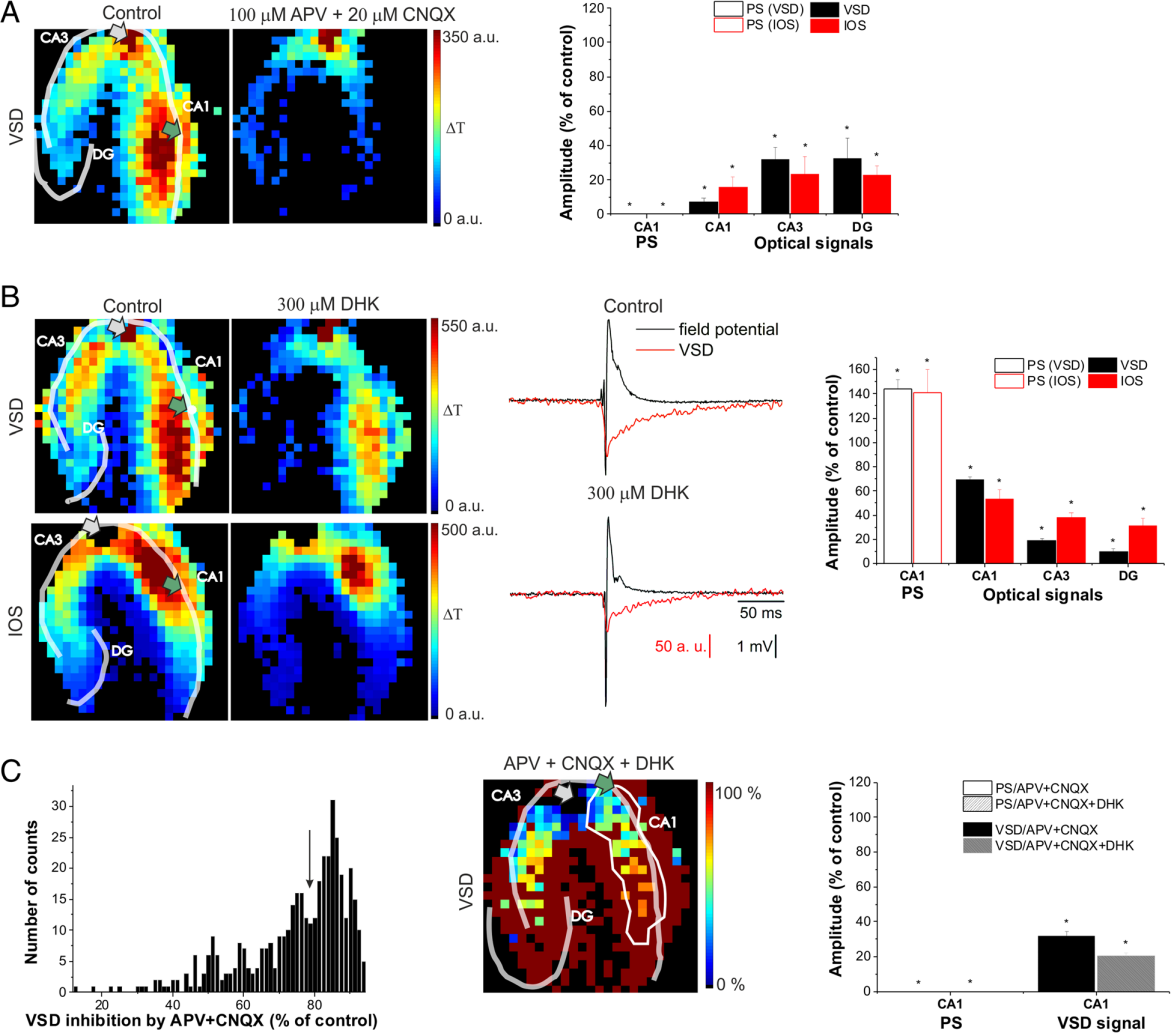
Contribution of glutamat*ergic* activity to VSD and IOS transients. **a**: (*Left*) Representative VSD amplitude maps under control condition and 100 μM APV + 20 μM CNQX application. (*Right*) Effect of 100 μM APV + 20 μM CNQX application on the population spike (PS) measured in the CA1 and on the average VSD and IOS amplitudes in the CA1, CA3 and DG regions. **b**: (*Left*) Representative IOS and VSD amplitude maps under control condition and 300 μM DHK application. (*Middle*) Time course of the field potential responses and VSD transients on the diode at the field potential recording site under control conditions and during 300 μM DHK application. (*Right*) Effect of 300 μM DHK on the PS measured in the CA1 and on the average VSD and IOS amplitudes in the CA1, CA3 and DG regions. **c**: (*Left*) Distribution of inhibitory effect of 100 μM APV + 20 μM CNQX application on the VSD amplitude of each diodes in % of control. The valley between the two peaks on the histogram is marked by an arrow. (*Middle*) Representative VSD inhibition map during 100 μM APV + 20 μM CNQX + 300 μM DHK application in % of control. Area identified as monosynaptically activated is contoured by white line. (*Right*) Effect of 100 μM APV + 20 μM CNQX + 300 μM DHK on the PS measured in the CA1 and on the average VSD amplitude in the monosynaptically activated area. On the optical signal amplitude maps transparent lines indicate the pyramidal cell layers (CA1 and CA3) and the granular cell layer of the DG. The positions of the stimulating and recording electrodes are marked by gray and green arrows, respectively. Asterisks indicate significant changes compared to control (P < 0.05, Mann–Whitney *U* test)

To assess the contribution of glutamat*ergic* processes to the VSD signal and IOS generation, we measured these signals in the presence of ionotropic Glu receptor antagonists and Glu transporter blocker. Simultaneous inhibition of 2-amino-3-(5-methyl-3-oxo-1,2-oxazol-4-yl)propanoic acid (AMPA)/kainate receptor by CNQX (6-cyano-7-nitroquinoxaline-2,3-dione, 20 μM) and NMDA receptor by APV (DL-2-amino-5-phosphonopentanoic acid, 100 μM) completely eliminated the PS amplitude (PS_IOS_: 0 ± 0 %, PS_VSD_: 0 ± 0 % of control) and significantly decreased the amplitude of IOS (16 ± 6 %, 23 ± 10 % 23 ± 6 % and 0 ± 0 % of control in the CA1, CA3 DG and at the FP recording site, respectively) and VSD signal (7 ± 2 %, 32 ± 7 %, 32 ± 12 % and 11 ± 11 % of control in the CA1, CA3 DG and at the FP recording site, respectively, Fig. 2a). There were no significant regional differences in either VSD or IOS, but the ratio of IOS and VSD signals was significantly different in all regions compared to control (Table 1), suggesting regional differences between the contribution of the ionotropic glutamat*ergic* receptors to IOS and VSD signals. In summary, the majority of all the three signals require preceding postsynaptic glutamat*ergic* activity.

**Table 1:** Differential effects of molecular target inhibitions on FP, VSD and IOS signals. Ratio of drug effects on optical signals and population spikes. Ratios were calculated by dividing the change in optical signal amplitude due to drug application (expressed in % of control) by the change in population spike or VSD amplitude (also expressed in % of control). Asterisks indicate significant changes compared to control (P < 0.05 Mann–Whitney U test). NA means not applicable (PS amplitude is 0 mV)

Molecular target (inhibitor)	VSD/PS ratio	IOS/PS ratio	IOS/VSD ratio	IOS/VSD ratio	IOS/VSD ratio
	CA1	CA1	CA1	CA3	DG
Control	0.90 ± 0.05	1.05 ± 0.02	1.09 ± 0.05	1.06 ± 0.06	1.03 ± 0.05
GluR receptors (CNQX + APV)	NA	NA	1.78 ± 0.30*	0.55 ± 0.22*	0.36 ± 0.13*
EAAT2 (DHK)	0.53 ± 0.04*	0.53 ± 0.08*	0.77 ± 0.16	1.98 ± 0.33*	5.68 ± 2.02*
KCC2 and NKCC1 (furosemide)	0.81 ± 0.10	0.43 ± 0.07*	0.45 ± 0.06*	0.46 ± 0.04*	0.32 ± 0.08*
NKCC1 (bumetadine)	0.93 ± 0.06	1.17 ± 0.07	0.94 ± 0.04	1.05 ± 0.05	1.09 ± 0.13
KCC2 (VU0463271)	0.53 ± 0.08*	0.53 ± 0.16*	1.00 ± 0.10	1.00 ± 0.10	0.91 ± 0.10
Anion channels (DIDS)	0.62 ± 0.05*	0.41 ± 0.07*	0.67 ± 0.08*	0.59 ± 0.08*	0.61 ± 0.11*
K_ir4.1_ (BaCl_2_)	0.65 ± 0.06*	0.8 ± 0.09*	1.29 ± 0.12	1.5 ± 0.12*	3.03 ± 0.75*
Na^+^/K^+^ ATPase (ouabain)	0.71 ± 0.09	0.63 ± 0.15	1.13 ± 0.12	1.10 ± 0.10	1.39 ± 0.10*

DHK (dihyrokainic acid, 300 μM), a selective blocker of the glial Glu transporter EAAT2 increased the PS amplitude (PS_IOS_: 141 ± 19 %, PS_VSD_: 143 ± 8 % of control, Fig. 2b) most probably due to the increased extracellular Glu concentration [40, 41]. Importantly, however, VSD and IOS amplitudes decreased in all regions of the hippocampus (VSD: 69 ± 2 %, 19 ± 1 %, 10 ± 2 % of control in the CA1, CA3 and in the DG, respectively; IOS: 54 ± 8 %, 38 ± 4 %, 31 ± 6 % of control in the CA1, CA3 and in the DG, respectively). Therefore, both IOS and the VSD signals were decoupled from neuronal firing, which is also indicated by the significant difference of the IOS/PS and VSD/PS ratios from the control (Table 1). These suggest that both VSD and IOS have a component that is directly related to the glial Glu transporter EAAT2, indicating that a large component of the optical signals has glial origin. This component may be attributed to the EAAT2 mediated glial cell swelling (in IOS signal), or glial depolarization via the EAAT2 (in VSD signal).

The fact that not only IOS, but also VSD decreased in the presence of DHK in parallel with the increasing PS amplitude could be the result that we measured the field potential locally in the CA1 *str. pyramidale* and compared it to summa VSD amplitude in the whole CA1. To address this issue we calculated the effect of DHK on the single diode where the FP recording electrode was located. We found that even at the location of the recording electrode the VSD signal decreased to 84 ± 3 % of control in the presence of DHK. This further substantiates that astroglial Glu uptake via EAAT2 is a major molecular determinant of the VSD signal even in the short (<5 ms) time-scale, which is in contrast to previous studies [6] that concluded astrocytic glutamate transport contribution only to the slow depolarizing response (>10 ms). We have also seen EAAT2 contribution to the slow depolarizing response as the integral of the VSD signal was significantly decreased to 51 ± 4 % of control in the presence of DHK, in agreement with the results of Kojima and coworkers [6]. It is to note that IOS was also decreased at the FP recording site (57 ± 11 % of control).

To confirm the strikingly fast contribution of the glial Glu transporter to VSD signals and to quantitatively determine its contribution, we measured the VSD inhibitory effect of DHK at the monosynaptically activated regions. Differentiating between mono- and polysynaptically activating areas is necessary because the increased extracellular Glu level leads to enhanced postsynaptic and polysynaptic activation potentially masking the EAAT2-mediated VSD signal components. In the monosynaptically activated regions, however, the contribution of iGluR- and EAAT2-mediated components can be directly measured and quantified. To quantitatively determine the contribution of EAAT2 transporter to the monosynaptic component of the VSD signal we sequentially applied iGluR inhibitors (CNQX and APV) followed by additional DHK (CNQX, APV and DHK). The mono- and polysynaptically activated regions were distinguished by the differential effect of iGluR inhibitors on the VSD signal (iGluR inhibition is stronger at polysynaptically activated areas due to the higher number of synapses involved in their activation). Indeed, the histograms of the iGluR inhibitor effects showed bimodal distribution (Fig. 2c). The area where APV and CNQX decreased the VSD signal by less than 78 % (the valley between the two peaks on the histogram) was considered to be monosynaptically activated (Fig. 2c). Afterwards, the effect of additional DHK was calculated in this region. We found that when DHK was added to the iGluR inhibitors the amplitude of the VSD signal was further decreased by 11 ± 1 % of control in this region (one way ANOVA, p = 0.0013, Fig. 2c) compared to iGluR inhibitors alone (32 ± 3 % of control, N = 6). Therefore a large part of the VSD signal (>10 %) is directly mediated by the glial Glu transporter EAAT2.

**Fig. 3:**
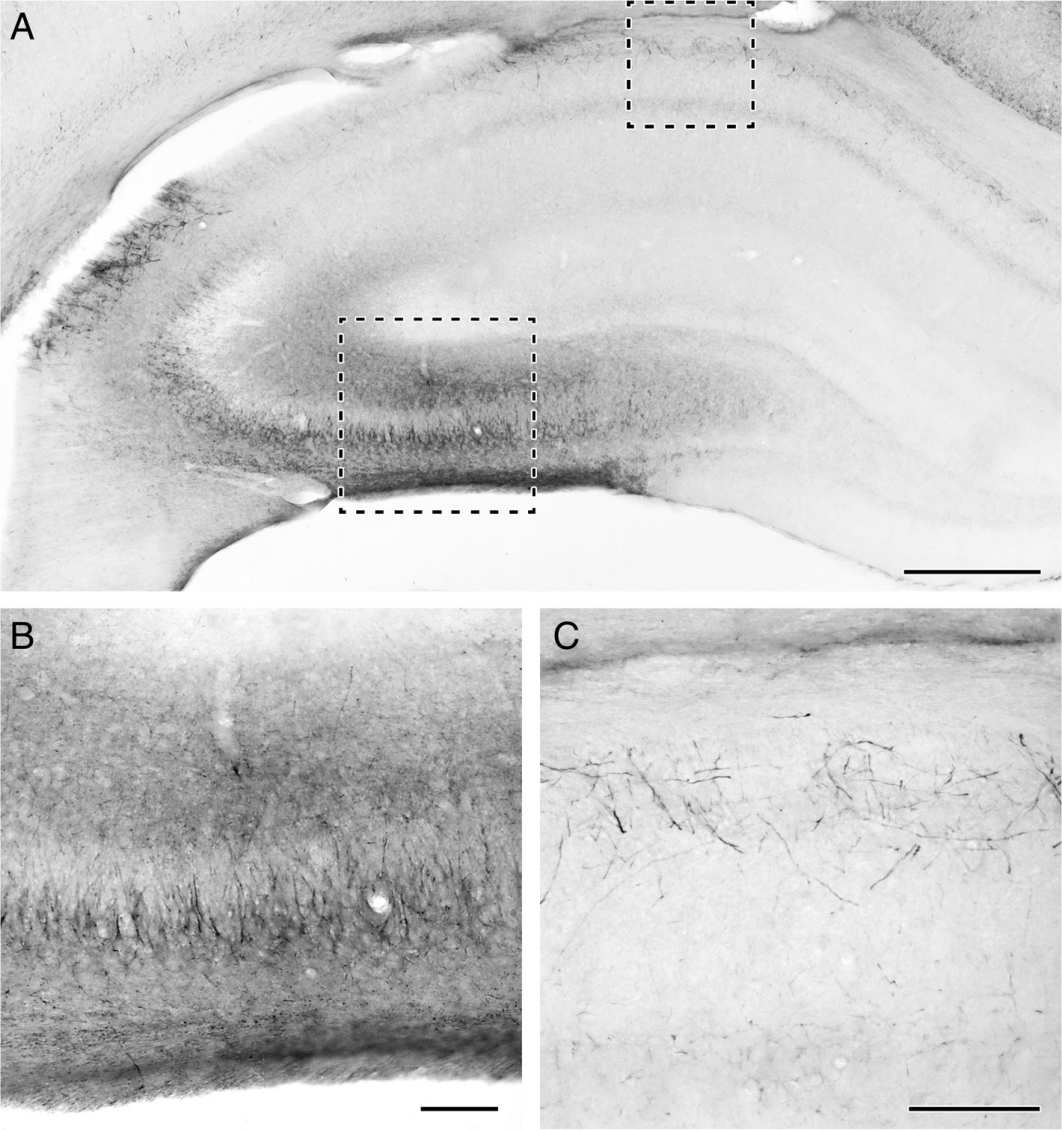
EAAT2 protein level is higher in the CA3 than in the CA1 region. **a**: The topographical distribution of EAAT2 is demonstrated in a coronal section. EAAT2 immunoreactivity is the most abundant in the CA3 region of the hippocampus and the *dentate gyrus*. However, EAAT2 immunoreactivity is also present in the CA1 region. **b**: A high magnification picture of the framed area in the CA3 region of the section shown in A is presented. Glial cell bodies are labeled in the pyramidal cell layer and adjacent part of *str. oriens*. Additional puncta-like labeling is seen throughout the *str. radiatum* and *str. oriens* representing astrocytic processes in these layers. **c**: A high magnification image of the framed area in the CA1 in A. EAAT2 immunolabeling is present in astrocytes distributed in *str. oriens* while faintly labeled cells are also present in *str. radiatum*. Scale bar = 500 μm for A and 100 μm for B and C

VSD in the CA1 and the CA3 regions decreased significantly more than IOS in the same regions. Interesting to note that contrary to the uneven IOS distribution, VSD amplitudes were significantly different in all regions (Fig. 2b). The most striking difference (50 ± 2 % of control) was found between the CA1 and the CA3 regions. In addition to the orto- vs. antidromic activation, these substantial differences can be attributed to regional differences in EAAT2 expression. To address this issue, immunohistochemistry measurements were performed. We found that EAAT2 protein level is higher in the CA3 region than in the CA1 region (Fig. 3) which can account for the regional differences of the EAAT2 mediated VSD signal component. These results are in agreement with previous studies showing that EAAT2 is more abundant in the CA3 and the *dentate gyrus*, compared to CA1 [42].

The effect of DHK was reversible as up to 70 % of control of the field response and optical signals were regenerated after 15 min washout. Therefore we can exclude the excitotoxic neuronal death.

In summary, in contrast to the Glu receptors, inhibition of the glial Glu transporter, EAAT2 strikingly discriminated between the field potential and both optical signals.

### The Na^+^/K^+^/Cl^−^ cotransporter NKCC1 does not differentiate VSD and IOS signals

NKCC1 is mainly expressed on astrocytes and transports Na^+^, K^+^ and Cl^−^ ions into the cell [43]. It is proposed to have a crutial role in the astrocytic K^+^ clearance [44]. The K^+^/Cl^−^ cotransporter KCC2 transports Cl^−^ and K^+^ ions out of the cells and is crucial for the maintenance of neuronal intracellular [Cl^−^] [45]. The precise balance between the activities of the two transporters is needed to maintain inhibitory GABA*ergic* signaling in the adult central nervous system [46]. We have previously showed [5] that the simultaneous inhibition of KCC2 and NKCC1 by furosemide (5 mM) had distinct effects on the PS and IOS. To determine whether IOS also differs from the spatial VSD signal, we examined the effect of furosemide on the VSD signal.

**Fig. 4:**
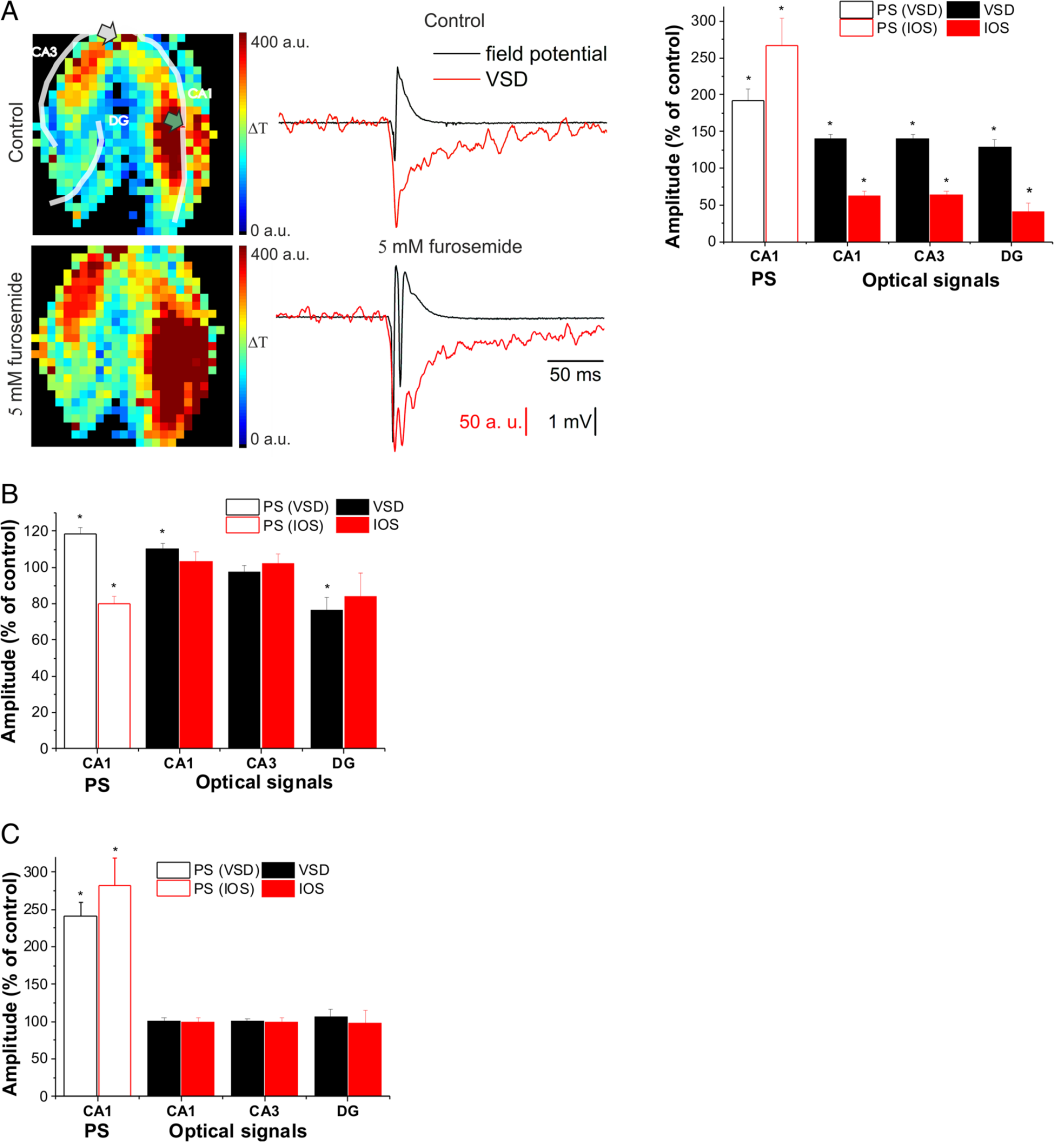
The Na^+^/K^+^/Cl^−^ cotransporter NKCC1 does not differentiate VSD and IOS signals. **a**: (*Left*) Representative VSD amplitude maps under control condition and 5 mM furosemide application. Transparent lines indicate the pyramidal cell layers (CA1 and CA3) and the granular cell layer of the DG. The positions of the stimulating and recording electrodes are marked by gray and green arrows, respectively. (*Middle*) Time course of the field potential responses and VSD transients on the diode at the field potential recording site under control condition and 5 mM furosemide application. (*Right*) Effect of 5 mM furosemide on the population spike (PS) measured in the CA1 and on the average VSD and IOS amplitudes in the CA1, CA3 and DG regions. **b**: Effect of 10 μM bumetanide on the PS measured in the CA1 and on the average VSD and IOS amplitudes in the CA1, CA3 and DG regions. **c**: Effect of 10 μM VU0463271 on the PS measured in the CA1 and on the average VSD and IOS amplitudes in the CA1, CA3 and DG regions. Asterisks indicate significant changes compared to control (P < 0.05, Mann–Whitney *U* test)

We observed that furosemide (5 mM) increased the PS amplitude (PS_IOS_: 267 ± 37 %, PS_VSD_: 193 ± 16 % of control) in the VSD and IOS experiments, likely due to the impaired GABA_A_ receptor mediated inhibitory function [47] or the reduced K^+^ driving force [48]. Parallel to the PS changes, the VSD signal amplitude also increased in all regions (140 ± 6 %, 140 ± 5 %, 129 ± 10 % and 159 ± 15 % of control in the CA1, CA3 DG and at the FP recording site, respectively; Fig. 4a). In contrast, IOS amplitude decreased in all regions (63 ± 6 %, 64 ± 5 %, 41 ± 12 % and 57 ± 21 % of control in the CA1, CA3, DG and at the FP recording site, respectively, Fig. 4a). Therefore, IOS, but not VSD decoupled from PS, indicated by the ratios of IOS and VSD signals that were significantly different in all regions compared to control (Table 1). Neither optical signal showed significant regional differences in drug effect, in line with the uniform expression pattern of the transporters [49]. The differential changes in IOS and VSD amplitudes confirm that a furosemide-sensitive molecular mechanism plays a crucial role in IOS generation.

After 15 min of washout major fractions of the PS and IOS was recovered, therefore neuronal death can be excluded.

Previous studies suggested that the effect of furosemide on IOS is mediated by the blockade of NKCC1 resulting in reduced glial swelling [8, 50] or reduced glial K^+^ clearance via the transporter [51]. However, bumetanide (10 μM), a specific antagonist of NKCC1 did not affect IOS amplitude in any region (104 ± 5 %, 102 ± 5 %, and 94 ± 3 % of control in the CA1, CA3 and DG, respectively; Fig. 4b), although the PS amplitude decreased to 80 ± 4 % of control. The decreased PS amplitude may be explained by the reported hyperpolarizing shift of the Cl^−^ reversal potential by inhibition of NKCC1 that leads to increased efficacy of GABA*ergic* inhibition [52]. IOS amplitude was also unaffected on the diode at the location of the recording electrode (94 ± 3 % of control).

In the presence of bumetanide the VSD signal increased in the CA1 to 110 ± 3 % of control, did not significantly change in the CA3 and at the FP recording site (97 ± 4 % of control and 106 ± 4 % of control, respectively) and decreased in the DG to 77 ± 7 % of control (Fig. 4b). Accordingly, the PS amplitude in the CA1 increased to 119 ± 4 % of control. The increased population spike amplitude can again be explained by impaired GABA_A_ receptor mediated inhibitory function [47] or the reduced K^+^ driving force [48]. Opposite to the IOS, VSD signal was not decoupled from neuronal activity by bumetanide, since PS amplitude in the CA1 and the VSD signal in the whole CA1 changed in a similar manner. Neither the optical signal/PS ratios nor the IOS/VSD ratio were significantly different from the control (Table 1). The differences between the changes of the PS amplitude in the case of IOS and VSD signal experiments could be accounted for the different stimulation protocol.

Since the specific antagonist of NKCC1 did not alter IOS/PS, VSD/PS and IOS/VSD ratios, we conclude that NKCC1 do not directly contribute to IOS and VSD signals in the rat hippocampal slice.

To further investigate the mechanism of action of furosemide, we applied VU0463271, a specific inhibitor of KCC2 (10 μM) [53]. In the presence of VU0463271 PS amplitude increased to a similar extent as in the case of furosemide application (PS_IOS_: 282 ± 37 %, PS_VSD_: 241 ± 18 % of control). This suggests that the furosemide induced increase in the PS amplitude is mediated by KCC2.

Despite the highly elevated PS amplitude, IOS (100 ± 6 %, 99 ± 7 % and 98 ± 18 % of control in the CA1, CA3 and DG, respectively; N = 5; Fig. 4c) and VSD (101 ± 4 %, 101 ± 3 % and 107 ± 9 % of control in the CA1, CA3 and DG, respectively; N = 7; Fig. 4c) amplitudes remained at control level in the CA1, CA3 and DG, and were only slightly increased at the FP recording site (IOS at the FP recording site 117 ± 7 %, VSD at the FP recording site 114 ± 6 %, of control). VU0463271 application did not alter the IOS/VSD ratios but the IOS/PS and VSD/PS ratios were significantly decreased compared to control (Table 1). There were no significant regional differences in drug effect in any of the optical signals, in line with the uniform expression pattern of the transporter [49].

The fact that VU0463271 could decouple neuronal activity from the optical signals implies that KCC2 takes part in the generation of both optical signals. However, VU0463271 application did not fully resemble the effects of furosemide, as it did not decrease the IOS and VSD signals and did not change IOS/VSD ratios. Therefore, furosemide may partly achieve its inhibitory effect by acting on proteins other than NKCC1 and KCC2, such as by inhibiting GABA_A_ receptors [54] or the Cl^−^/HCO3^−^ exchanger [55]. Additional inhibition of GABA_A_ receptor by furosemide could also explain the increase of VSD amplitude by furosemide, but not by VU0463271. Similarly, inhibitory action of furosemide on the Cl^−^/HCO3^−^ exchanger might explain why IOS was decreased in the presence of furosemide, because besides KCC2 the Cl^−^/HCO3^−^ exchanger might also play a role in cell swelling [56] and it is expressed in the hippocampus [57]. However, since the inhibition of the GABA_A_ receptor by picrotoxin enhanced IOS, the non-specific effect of furosemide on this receptor could not be responsible for IOS decrease. Alternatively, another possible explanation is that a synergistic effect arises when NKCC1 and KCC2 are simultaneously blocked.

In summary the role of NKCC1 can be excluded from the generation of optical signals. The results suggest the KCC2 contributes to both optical signals, but its inhibition by a specific antagonist cannot fully match the effect of furosemide.

### Inhibition of glial potassium clearance differently affects VSD and field potential

Previous studies concluded that IOS mainly reflects glial K^+^ clearance via the NKCC1 transporter [50, 58]. This conclusion was drawn based on the inhibitory effect of furosemide, an unspecific blocker of both NKCC1 and neuronal KCC2. Since we excluded the role of NKCC1 in IOS generation and showed that furosemide likely acts on the neuronal potassium influx transporter KCC2, the question arises whether potassium clearance by other proteins significantly contributes to IOS. Moreover, potassium clearance was also suggested to contribute to the VSD signal in skate brain slice [59]. Therefore, we investigated the role of major potassium clearance routes, the K_ir4.1_ potassium channel and the glial Na^+^/K^+^ ATPase [60–62] in IOS and VSD signal generation.

**Fig. 5:**
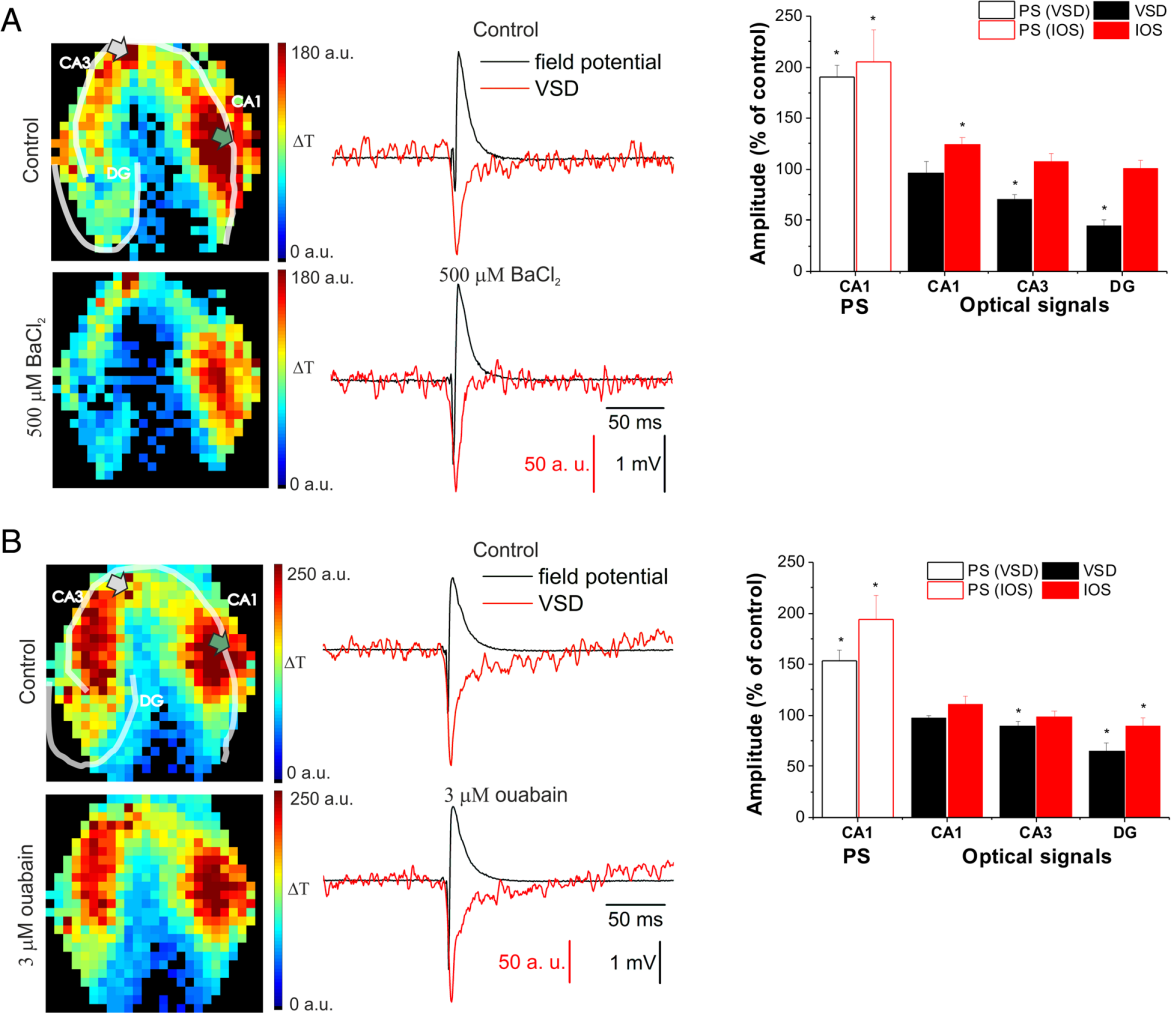
Inhibition of glial K^+^ clearance only slightly affects IOS, but significantly decreases VSD transients. **a**: (*Left*) Representative VSD amplitude maps under control condition and 500 μM BaCl_2_ application. (*Middle*) Time course of the field potential responses and VSD transients on the diode at the field potential recording site under control condition and 500 μM BaCl_2_ applications. (*Right*) Effect of 500 μM BaCl_2_ on the population spike (PS) measured in the CA1 and on the average VSD and IOS amplitudes in the CA1, CA3 and DG regions. **b**: (*Left*) Representative VSD amplitude maps under control condition and 3 μM ouabain application. (*Middle*) Time course of the field potential responses and VSD transients on the diode at the field potential recording site under control condition and 3 μM ouabain application. (*Right*) Effect of 3 μM ouabain on the PS measured in the CA1 and on the average VSD and IOS amplitudes in the CA1, CA3 and DG regions. On the VSD signal amplitude maps transparent lines indicate the pyramidal cell layers (CA1 and CA3) and the granular cell layer of the DG. The positions of the stimulating and recording electrodes are marked by gray and green arrows, respectively. Asterisks indicate significant changes compared to control (P < 0.05, Mann–Whitney *U* test)

Due to the lack of a specific inhibitor for K_ir4.1_ potassium channel, its role was addressed by the non-specific blocker BaCl_2_ (500 μm) [63, 64] on the FP, IOS and VSD signals. BaCl_2_ increased PS amplitude (PS_IOS_: 191 ± 11 %, PS_VSD_: 206 ± 31 % of control, Fig. 5a). The IOS amplitude also increased in the CA1 region and at the FP recording site (125 ± 7 % and 119 ± 8 % of control) but did not change in the CA3 and in the DG (108 ± 7 % and 101 ± 8 % of control for CA3 and DG, respectively; Fig. 5a).

VSD signal was not increased by BaCl_2_ in the whole CA1 region (97 ± 11 % of control), but it was increased at the recording electrode site (125 ± 5 % of control). VSD signal was significantly decreased in the CA3 and in the DG (71 ± 5% and 45 ± 6 % of control for CA3 and DG, respectively; Fig. 5a), suggesting region specific contribution of astrocytes to the VSD signal.

Importantly, besides the K_ir4.1_ potassium channel, BaCl_2_ also inhibits K_ir2.x_ potassium channels [65, 66], K_ATP_ potassium channel [67] and KCNQ1 potassium channel [68]. However, these channels are localized on neurons, therefore their inhibition would result in neuronal depolarization and consequently increased VSD signal amplitude. The observation that VSD and PS amplitudes change in the opposite direction suggests that BaCl_2_ acts on the astrocytic potassium channels. Since K_ir4.1_ potassium channel is the most abundantly expressed potassium channel on astrocytes [69], BaCl_2_ most likely acts via K_ir4.1_ potassium channel inhibition.

Besides K_ir4.1_, the Na^+^/K^+^ ATPase is also responsible for the glial K^+^ clearance [62]. Moreover, it was shown that the glial Na^+^/K^+^ ATPase isoform plays a major role in Schaffer-collateral stimulation evoked extracellular K^+^ clearance [60]. Since the neuronal α1 isoform of the rat Na^+^/K^+^ ATPase is very insensitive to its inhibitor ouabain (IC_50_ > 1 mM) [70–72], we could assess the contribution of the more ouabain sensitive [73] glial α2 Na^+^/K^+^ ATPase isoform by low concentration (3 μM, [74]) ouabain. Ouabain application evoked changes in PS, IOS and VSD amplitudes very similar to those observed for BaCl_2_ (Fig. 5b). PS amplitude was increased (PS_IOS_: 154 ± 10 %, PS_VSD_: 194 ± 24 % of control). IOS amplitude was not significantly increased in the whole CA1 region (111 ± 8 % of control), but was significantly increased on the diode at the FP recording electrode site (126 ± 12 % of control). IOS amplitude did not change in the CA3 and DG regions (99 ± 5 % and 90 ± 7 % of control for CA3 and DG, respectively). VSD signal also showed similar pattern as IOS. In the whole CA1 the VSD signal amplitude remained at control level (98 ± 3 % of control), but at the FP recording electrode site VSD amplitude increased (116 ± 5 % of control). In the CA3 and the DG regions, the VSD amplitude significantly decreased (90 ± 4 % and 65 ± 9 % of control for CA3 and DG, respectively; Fig. 5b), similarly to the BaCl_2_ application_._

Conclusively, glial K^+^ clearance via the K_ir4.1_ potassium channel and the glial Na^+^/K^+^ ATPase can only be a minor contributor to the IOS and VSD signals.

IOS can also originate from the increased metabolic activity of glial cells. To determine if the enhanced mitochondrial citric acid cycle activity contribute to IOS or VSD, we tested the effect of fluorocitric acid (322 μM) the blocker of the aconitase enzyme [75, 76]. Since fluorocitric acid is available as a Ba^2+^ salt, we have chosen its concentration to match the concentration of Ba^2+^ applied in the previous experiments. In the presence of fluorocitric acid IOS amplitude increased in the CA1 region and at the FP recording site (116 ± 10 % and 118 ± 8 %, respectively) but remained at control level in the CA3 and in the DG (98 ± 8 % and 84 ± 14 % of control in the CA3 and DG, respectively). At the FP recording site the VSD signal amplitude remained at control level (82 ± 18 % of control), but in the CA1, CA3 and the DG VSD amplitude was significantly decreased (to 68 ± 11 %, 50 ± 10 % and 53 ± 17 % of control in the CA1, CA3 and DG, respectively). Since the results for the fluorocitric acid were not significantly different from the results of BaCl_2_ (p < 0.05, one way ANOVA), we conclude that fluorocitric acid does not have any effect on IOS and VSD signals.

### Assessing the contribution of anion homeostasis to the optical signals

**Fig. 6:**
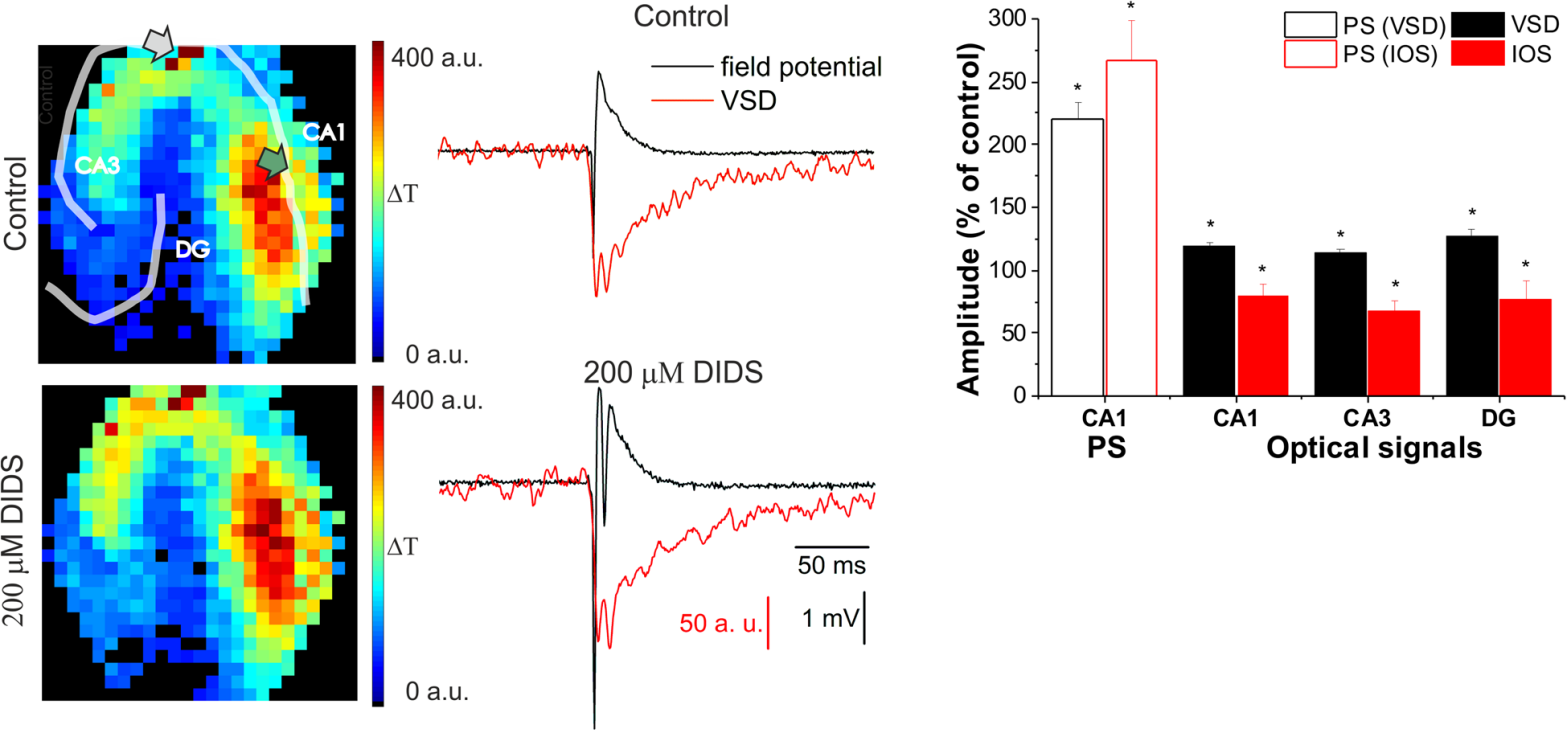
DIDS enhances VSD and decreases IOS transients. (*Left*) Representative VSD signal amplitude maps under control condition and 200 μM DIDS application. Transparent lines indicate the pyramidal cell layers (CA1 and CA3) and the granular cell layer of the DG. The positions of the stimulating and recording electrodes are marked by gray and green arrows, respectively. (*Middle*) Time course of the field potential responses and VSD transients on the diode at the field potential recording site under control condition and 200 μM DIDS application. (*Right*) Effect of 200 μM DIDS on the population spike (PS) measured in the CA1 and on the average VSD and IOS amplitudes in the CA1, CA3 and DG regions. Asterisks indicate significant changes compared to control (P < 0.05 Mann–Whitney *U* test)

We have previously shown that the general anion channel antagonist DIDS (200 μM) decoupled neuronal activity from IOS [5]. However, it is more appropriate to compare the IOS signal to the spatial VSD instead of the locally measured FP. Therefore we compared DIDS effect on IOS and VSD. Population spike amplitude was increased in both measurements (PS_IOS_: 267 ± 32 %, PS_VSD_: 220 ± 13 % of control). VSD signal amplitude increased in all regions of the slice (120 ± 3 %, 114 ± 3 %, 127 ± 5 % and 119 ± 5 % of control for CA1, CA3 DG, and at the FP recording site respectively, Fig. 6) with no significant regional differences. IOS amplitude also uniformly decreased in all regions (80 ± 9 %, 67 ± 9 % 78 ± 15 % and 75 ± 6 % of control for CA1, CA3, DG and at the FP recording site, respectively). Similarly to furosemide application, the ratio of IOS and VSD signals was significantly different in all regions compared to control (Table 1), suggesting that the target of DIDS is a uniformly expressed protein in the slice. Both IOS and the VSD changed differently than the PS, indicated by the significant difference of the IOS/PS and VSD/PS ratios from the control (Table 1). DIDS shows a behavior similar to furosemide, indicating that the two compounds may have a common target molecule.

## Discussion

VSD and IOS imaging are widely used methods for mapping neural activity both *in vitro* and *in vivo*. Although these signals pertain spatiotemporal spreading of stimulus evoked neural activity, associated cellular and molecular activities as well as the involvement of neuro-glia coupling are not concluded yet. We addressed these issues by fast imaging in combination with molecular profiling of IOS and VSD transients evoked by stimulating Schaffer collaterals in rat hippocampal slices. For comparison, FP was also recorded from area CA1.

**Fig. 7:**
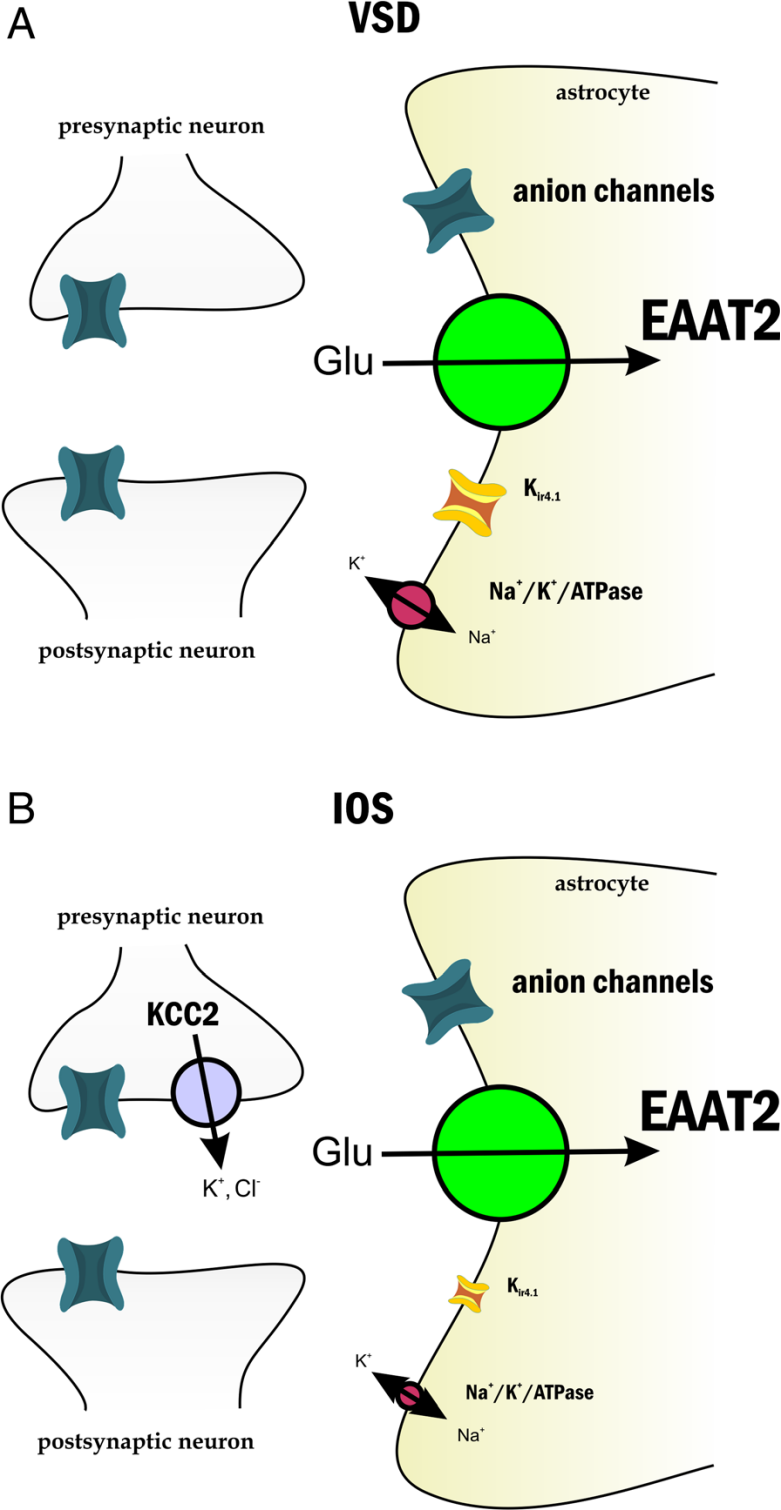
Molecular dissection of processes underlying spatiotemporal IOS and VSD generation. **a**: Neuronal and glial proteins underlying VSD mechanism. First, stimulus of the Schaffer collaterals depolarizes neurons via Glu receptor activation. The released Glu almost instantly activates glial EAAT2, therefore the first 5 ms of the VSD signal represents both neuronal and glial depolarization. Neuronal depolarization leads to additional Glu release and the elevation of extracellular [K^+^] and alteration of extracellular [Cl^−^]. The elevated extracellular [K^+^] activates glial K_ir4.1_ channel and Na^+^/K^+^ ATPase which are responsible for the late VSD response. The altered extracellular [Cl^−^] leads to the activation of non-specific anion channels and transporters which also contribute to the VSD signal. **b**: Neuronal and glial proteins underlying IOS mechanism. First, neurons in the pyramidal layer are activated by Schaffer collateral stimulation leading to swelling of their soma, followed by swelling of glial cells in the dendritic region. Glial activity related IOS appears as the consequence of neuronal activation. Neurons induce glial IOS through altered extracellular [K^+^] and [Cl^−^] concentration by depolarization, by their KCC2 cotransporter and by the release of glutamate. IOS is mediated on glial cells via the EAAT2, anion channels and transporters, K_ir4.1_ channel and Na^+^/K^+^ ATPase. Proteins underlying VSD or IOS mechanism are sized in accordance with their contribution to IOS and VSD genesis

Major findings are summarized as follows: *i*) glutamate clearance by astroglial glutamate transporter EAAT2 is a major component of both IOS and VSD transients (Fig. 7); *ii*) EAAT2 contributes to VSD generation in very short time scale (<5 ms); *iii*) regiospecific contribution of astrocyte activity to VSD in the order of DG > CA3 > CA1 correlates with the EAAT2 localization pattern; *iv*) activities of neuronal K^+^/Cl^−^ cotransporter KCC2, non-specific anion channels, astroglial K^+^ clearance through K_ir4.1_ channel and Na^+^/K^+^ ATPase also contribute to the spatiotemporal IOS and VSD signaling (Fig. 7); *v)* IOS and VSD signals are critically dependent on voltage-dependent Na^+^ channel activity, GABA*ergic* disinhibition and postsynaptic glutamat*ergic* activation, reflecting eventual neuronal origin; *vi*) despite their diverse spatiotemporal spreading, the IOS and VSD amplitudes linearly correlate to the PS amplitude.

### Astrocytic Glu uptake shapes spatiotemporal spreading of VSD and IOS transients

Blockade of IOS and VSD signals by tetrodotoxin and substantial disinhibitory enhancements by picrotoxin support causal relationship between spatiotemporal spreading of neuronal excitation and VSD/IOS transients. IOS and VSD transients have previously been suggested to reflect postsynaptic activation of neurons [8, 23, 77–80]. In accordance, we found that both IOS and VSD transients were significantly decreased in the presence of ionotropic glutamate receptor AMPA/kainate and NMDA antagonists.

Importantly, however, we also identified significant astrocytic components in both the VSD and IOS signals. Astrocytes have a key role in glutamat*ergic* neurotransmission, since Glu is mainly taken up by astrocytes [81–83]. Therefore astroglial Glu uptake can be a plausible component process of the spatiotemporal optical signals [6, 84]. We have recently reported [5] that DHK, a selective blocker of the astroglial Glu transporter EAAT2 had distinct effects on IOS and PS measures of neural activity, indicating a direct role for astrocytic Glu uptake mediated cell swelling in IOS generation. Surprisingly, DHK differentiated not only between IOS and PS, but also between VSD and PS. To confirm this unexpected result, we also determined the DHK effect not only regarding the whole CA1 region, but also on the single photodiode at the FP recording electrode site. The direct comparison of the VSD and FP transients from the same location still showed distinct DHK effects on VSD absorbance and FP traces, excluding the possibility that spatial averaging of the VSD transients introduced the EAAT2-sensitive component.

The effect of DHK on the VSD signal and PS amplitude can be either direct by decreasing glial EAAT2 transporter currents or indirect by increasing interneuron firing rate [85] or desensitizing Glu receptors. Indirect effect of DHK, however, can be excluded since PS amplitude increased in the presence of DHK, indicating enhanced neuronal activity due to increased extracellular [Glu]. Therefore, we can conclude that the EAAT2 transporter directly contributes to the generation of VSD signal.

EAAT2 is mainly expressed on glial cells [86–90], and glial cells are responsible for the majority of Glu uptake [83, 88]. It should be noted, however, that EAAT2 expression was also demonstrated on neuronal axon terminals and axons [91–93]. However, in contrast to the synapse-facing glial EAATs, neuronal EAAT2 is located extrasynaptically [94], therefore its activation requires Glu to escape the synapse. Accordingly, significant capacity of neuronal Glu uptake could only be observed when exogenous substrates were applied [92, 95] and it is concluded that neuronal EAAT2 do not play significant role in the removal of synaptically released Glu [83, 96]. In addition, we demonstrated the inhibitory effect of DHK on VSD in a very short time scale (<5 ms) that probably does not allow Glu escape from the synapse. Therefore, the neuronal EAAT2 is less likely to contribute to the VSD signal in the observed (>10 %) extent. Moreover, inhibition of neuronal Glu (and coupled sodium) uptake would lead to hyperpolarization of neurons which is in contrast to the measured increase in the PS amplitude. Conclusively, we propose that it is the glial EAAT2 that plays a major role in VSD generation.

We have demonstrated that DHK decreases VSD amplitude that appears within 5 ms from the stimulus, suggesting glial contribution in a very short time scale. Despite glial cells are generally considered to be slow responding cells that operate on longer time scales [97], several studies have demonstrated that glial Glu transporter currents do appear within this very short (<5 ms) interval after synaptic activation [94, 98, 99]. In addition, neurotransmitter transporters are slowly transporting their substrates, again arguing against the quick contribution of EAAT2 in VSD generation. However, despite the slow transport cycles, Glu transporters are able to generate quick currents due to their high density. The number of EAAT2 proteins in a single CA1 synapse is 3–5 times higher than the number of Glu molecules in a synaptic vesicle [87]. Therefore even one transport cycle can be sufficient to remove all the released Glu molecules.

In contrast to our results, previous studies showed only significantly delayed astrocytic contribution to VSD signaling (>10 ms) that was considered as a depolarization response [6]. It should be noted that we have also seen this delayed astrocytic contribution to the VSD signal in the presence of DHK. The apparent conflict between these results may be attributed to the different locations the VSD signals were acquired relative to the stimulus site. Kojima et al. measured VSD responses in the close vicinity of the stimulation electrode, while we detected VSD signals at several hundred μm away from the stimulation electrode. In our experiments, the signals on diodes close to the stimulation site in the CA3 could not be evaluated due to the large artefacts likely caused by vessel dilation.

Another important issue regarding the EAAT2-mediated components of the VSD signal is why it does not appear on the FP responses, despite the common parameter (membrane potential fluctuations) probed by the two methods. We propose that this apparent discrepancy may be due to the expression profile of EAAT2. EAAT2 is located on perisynaptic astroglial processes [100] and the overall surface of these processes largely exceeds the membrane area of the soma and main processes [101]. VSD is bound to the cell membrane [102] over the full membrane surface, therefore it can adequately probe the membrane potential changes of the astrocyte processes, which are hardly detectable even by patch clamp recording [98, 103].

By identifying the monosynaptically activated region, we also quantified the contribution of glial EAAT2 to the generation of the VSD transients. The EAAT2-sensitive component was found to be a major portion (>10 %) of the total VSD signal. These findings provide evidence for the critical role for astrocytes and neuro-glia interactions [104–106] in the generation of VSD transients for the first time.

### Contribution of anion homeostasis to VSD and IOS signals

The general anion channel antagonist DIDS increased PS amplitude in the CA1 by 120 ± 13 %, but enhanced VSD only by 20 ± 3 %. The differential effect of DIDS suggests that its target proteins contribute not only to IOS [5], but also to the VSD signal (this work). Since DIDS may act on multiple targets, including the mitochondrial inner membrane anion channel [107], K_v4.2_ and K_v4.3_ potassium channels [108], chloride channels [109], transient receptor potential cation channel subfamily C member 4 (TRPC4), transient receptor potential cation channel subfamily M member 4 (TRPM4) [110], transient receptor potential cation channel subfamily V member 1 (TRPV1), RAD51 recombinase [111], Na^+^/CHO_3_^−^ cotransporter [112] and Cl^−^/HCO3^−^ exchanger [113], it is difficult to determine which target or targets are responsible for its effect on the optical signals. Inhibition of neuronal TRPM4, TRPC4, K_v4.2_ or K_v4.3_ potassium channels by DIDS would lead to decreased depolarization and consequently decreased PS amplitude. Therefore, these target molecules are not likely to mediate DIDS effect. TRPV1 currents are potentiated by DIDS, but the activity of this channel is associated with synaptic depression [114], thus TRPV1 may also be excluded from the potential mediators. Since IOS has been shown to be sensitive to the alteration of the chloride gradient [79], the most probable target molecules of DIDS are the anion channels, Na^+^/CHO_3_^−^ cotransporter or Cl^−^/HCO3^−^ exchanger. Notably, these proteins are expressed on glial cells [43, 115–117], which can also explain why VSD is decoupled from neuronal activity in the presence of DIDS.

Since DIDS effect on FP and VSD signals was comparable to furosemide, our findings may conjecture furosemide and DIDS sharing a common target involved in IOS and VSD generation.

### Contribution of astrocytic K^+^ clearance to VSD and IOS transients

By addressing the role for K_ir4.1_ potassium channel in VSD and IOS transients with BaCl_2_, a non-specific inhibitor of the K_ir4.1_ potassium channel we observed similar effects on amplitudes of both PS and optical signals in the CA1. However, despite of the increased PS and VSD amplitudes, the integral of the VSD transient significantly decreased in the CA1 due to the altered VSD curve shape. In addition, the IOS/VSD amplitude ratio also suggested distinct contributions of K_ir4.1_ activity to IOS and VSD signals in the CA3 and DG regions. Similarly to the K_ir4.1_ channel inhibition, blockade of the astroglial Na^+^/K^+^ ATPase resulted in region-specific changes in IOS/VSD ratios. Since K_ir4.1_ channel and astroglial Na^+^/K^+^ ATPase inhibition did not decouple the PS and optical signal amplitudes, they likely do not play major roles in IOS and VSD generation. The region-specific IOS/VSD ratios and the distinct effect of K_ir4.1_ channel inhibition on the PS amplitude and VSD integral, however, suggests that they play minor role in VSD and IOS signals. It is to note that their contribution can also be indirect via interfering astrocyte glutamate uptake [88].

## Conclusion

Molecular dissection of VSD and IOS transients combined with simultaneous, time-aligned field potential recordings have highlighted the role of various membrane proteins in the generation of the two widely used optical readout of neural activity. Importantly, both signals have been shown to be rooted from a mixture of neuronal and glial processes. Contrary to the general view of IOS that attributes signal genesis mostly to glial cell swelling, we identified a significant neuronal component mediated by the K^+^/Cl^−^ cotransporter KCC2. Surprisingly, our findings also suggest that the interpretation VSD signals as spatially extended field potentials may not be relevant*,* since the VSD transients contain a significant astroglial component that is not present in the field potentials. Molecular entities mediating this VSD-specific astrocytic component have been identified as the EAAT2 glutamate transporter (major) and possibly the K_ir4.1_ channel as well as the astroglial Na^+^/K^+^ ATPase (minor). Therefore, neither VSD imaging can be considered as a predominantly neuronal signal, nor IOS should be associated with primarily glial processes. Instead, both optical signals obey astroglial responses to neuronal activity. These findings constitute neuro-glia communication as the origin of spatiotemporal optical signals within the brain.

## Methods

### Ethical approval

Animals were kept and used in accordance with the European Council Directive of 24 November 1986 (86/609/EEC), the Hungarian Animal Act, 1998. All experiments involving animals were done by the approval of the Animal Testing Committee of the Research Centre for Natural Sciences, Hungarian Academy of Sciences and by the approval of the Ministry of Agriculture and Rural Development, Hungary. All efforts were made to reduce animal suffering and the number of animals used.

### Chemicals

Picrotoxin, DIDS, fluorocitric acid, barium chloride and furosemide were purchased from Sigma-Aldrich Co. (St. Lois, MO, USA). Tetrodotoxin (TTX), DHK, ouabain, VU0463271 and bumetanide were purchased from Tocris Bioscience (Bristol, UK). CNQX and APV were purchased from Abcam Biochemicals (Cambridge, UK).

### Brain tissue slices

Transverse, 400 μm thick hippocampal-entorhinal cortex slices from juvenile (P21-50) male Wistar rats (Toxicoop, Budapest, Hungary) were prepared in modified artificial cerebrospinal fluid (ACSF, in mM: 75 sucrose, 87 NaCl, 2.5 KCl, 1.25 NaH_2_PO_4_, 7 MgSO_4_, 0.5 CaCl_2_, 25 NaHCO_3_, 25 glucose, continuously bubbled with 95% O_2_ + 5% CO_2_ gas mixture) at 4°C, as described before [118]. Slices were incubated in an interface-type chamber that was continuously circulated by ACSF for 1 h at 37°C (followed by incubation at room temperature) before performing the experiments in a submerged type recording chamber perfused with carbogen gas-saturated ACSF (containing in mM: 129 NaCl, 10 glucose, 3 KCl, 1.25 NaH_2_PO_4_, 1.8 MgSO_4_, 2 CaCl_2_, 21 NaHCO_3_, pH 7.4, 36 °C) at a rate of 3–4 ml/min. All drug effects were measured in at least two animals.

### Voltage-sensitive dye staining

For VSD imaging slices were stained for 10 min at room temperature in 1ml bubbled ACSF containing 0,307 μM voltage-sensitive absorbance dye NK3630 (RH482, Hayashibara Biochemical Laboratories, Okayama, Japan) [119, 120] in a custom designed interface chamber (1,2 ml total volume). Then slices were washed three times in the small interface chamber by 1 ml ACSF and kept in the dark for 1 h before the experiment. NK3630 dye was only applied for VSD measurements except one case, when the spatial extent of VSD and IOS were compared in the same slice (Fig. 1a).

### Schaffer collateral stimulation

Single square wave pulses of 0.2 ms duration were delivered through a bipolar tungsten electrode (75 μm diameter, Microprobes for Life Science), placed in the trajectory of the Schaffer collaterals in the *str. radiatum* of the CA3 region. Each stimulus was preceded by a 200 ms long delay period for baseline calculations. Stimulation intensity was set to evoke half maximal field response (40–50 V). Different stimulation protocols were used to evoke IOS (10 stimuli/20 Hz) and VSD (single pulses) signals. The differences between the stimulation protocols were made to clearly discriminate the two, spectrally overlapping signals. IOS cannot be evoked by one stimulus [5], therefore a single pulse specifically evokes VSD. VSD, however, cannot be reliably measured using a stimulus train, because it lasts longer than the interstimulus interval (50 ms), therefore its amplitude is summed during the train. In all experiments VSD was evoked by a single stimulus while the IOS was evoked by a 20 Hz stimulus train. When comparing the spatial extent of VSD and IOS, optical signals were recorded from the same slice, but were evoked by different stimulus protocols (Fig. 1a). Three or five trials were taken in every 10 min for IOS and VSD measurements, respectively.

### Optical recording

Optical signals were detected by a 464-element photodiode array (PDA) detector having temporal resolution of 0.6 ms, making it achievable to align the optical signals with the simultaneously measured FP recordings.

**Fig. 8:**
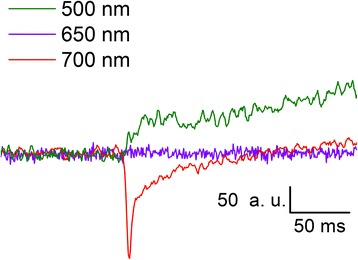
Spectral characteristics of recorded VSD. VSD signals detected at different illumination wavelengths

For both type of optical measurement, IOS and VSD, slices were illuminated with a halogen lamp source (maximum intensity: 100 W) passed through a band-pass filter (700 ± 40 nm, Chroma Technology, Rockingham, VT). For the validation of the VSD signal a 500 ± 40 nm (Chroma Technology, Rockingham, VT) and a 650 ± 3 nm (Knight Optical (UK) Ltd, Harrietsham) band-pass filters were also used. As expected [120], no VSD signal could be observed at 650 nm, a small positive amplitude signal was measured at 500 nm and the highest amplitude signal was detected at 700 nm (Fig. 8).

The transmitted light was collected by a Wutech H-469IV photodiode array that is part of a Redshirt Imaging integrated Neuroplex II imaging system, mounted on the front port of an Olympus BX51WI microscope (10x objective, 1.6 kHz sampling frequency, 500× amplification, 0.5 Hz high-pass filtered [119]). Each diode in the array corresponds to an approximately 70 × 70 μm square area. Data were acquired using the Neuroplex software.

### Field potential recording

Field potential recording electrodes (2–5 MΩ) were filled with ACSF and placed in the CA1 pyramidal cell layer. Recordings were performed with a two-channel Multiclamp 700A amplifier (Axon Instruments, Foster City, CA, USA). Traces were low-pass filtered at 2 kHz and digitized at 10 kHz with a Digidata 1320A A/D converter (Axon Instruments, Foster City, CA) controlled by a computer running pClamp8 (Axon Instruments, Foster City, CA, USA).

### Drug application

After recording at least two control trials, drugs were perfused in the recording chamber at a rate of 3–4 ml/min. For evaluating drug effects, the trials were taken after 5 min of drug application.

The stability of the electrophysiological and optical signals were tested in the timeframe of the drug application experiments. Control measurements were performed, in which no drug was added, but the parameters of the signals were calculated in the same way as in the case of drug application. IOS amplitude was slightly, but significantly higher in the CA1 and CA3 regions after 3 control trials (106 ± 4 %, 108 ± 3 % of control in the CA1, and in the CA3, respectively N = 5, p < 0.05), while the field potential and VSD amplitudes showed no changes (N = 8). Drug effects were considered significant if the changes exceeded two times the baseline change.

In the experiments in which ouabain was applied, slices showing <70 % of the control FP amplitude were excluded from further data analysis.

### Immunohistochemistry

Three rats were deeply anesthetized and perfused transcardially with 150 ml saline followed by 300 ml of ice-cold 4 % paraformaldehyde prepared in 0.1 M phosphate buffer at pH 7.4 (PB). Brains were removed and postfixed in 4 % paraformaldehyde for 24 h and then transferred to PB containing 20 % sucrose for 2 days. Serial coronal sections were cut at 50 μm on a sliding microtome from a block that included the hippocampus. Sections were collected in PB containing 0.05 % sodium-azide and stored at 4 °C.

Every fourth free-floating brain section was immunostained for EAAT2 using a monoclonal mouse anti-EAAT2 antiserum (mouse anti-EAAT2, cat# ab77039, Abcam, Cambridge, UK). This antiserum (1:500) was applied for 48 h at room temperature followed by incubation of the sections in biotinylated donkey anti-mouse secondary antibody (1:1000 dilution; Jackson ImmunoResearch, West Grove, PA) for 1 h and then in ABC complex (1:500; Vector Laboratories, Burlingame, CA) for an additional 1 h. Subsequently, EAAT2 immunoreactivity was visualized by incubating the sections in 0.02 % 3,3-diaminobenzidine (DAB; Sigma), 0.08 % nickel(II) sulfate, and 0.003 % hydrogen peroxide in PB. Finally, the sections were mounted, dehydrated and coverslipped with Cytoseal 60 (Stephens Scientific, Riverdale, NJ).

### Primary data processing and data analysis

The population spike (PS) amplitude was calculated according to Anderson and Collingridge [121] as the amplitude from the PS onset to the intersection with a tangent line drawn between the PS onset and the PS offset. For the IOS experiments, the amplitudes of the 10 evoked PS responses in the stimulus train were summed and compared to the sum IOS amplitude.

The intensity values on each diode were divided by the resting (background) light intensity on the same pixels to reduce regional differences in scattering properties or staining level. IOS and VSD signals were smoothed with local regression method using weighted linear least squares and a 2nd degree polynomial model [122]. The detected optical signal peaks were accepted if the signal to noise ratio has exceeded 5.

Drug effects were quantified by calculating the amplitude (difference between the peak intensity after the stimulus and the average of baseline intensity level recorded before the stimulus) and integral (the area under curve from the onset to the point when the signal reached the baseline level) of IOS and VSD transients. Regional IOS and VSD parameters were calculated for the whole CA1, CA3 and *dentate gyrus* by summing amplitudes on all diodes in the selected region. Diodes close (<100 μm) to the stimulating electrode were excluded from the analysis due to the large movement artifact following the stimulus.

To characterize the spreading pattern of the optical signal, the time point where the IOS or VSD curve reaches 50 % of its amplitude was also calculated (referred to as 50 % delay). We have chosen this method because the location of maxima of the optical signals (especially in the case of IOS) could not always be reliably identified. Spreading velocity of the VSD was estimated by dividing the distance between two diodes by the difference between the 50% delay points of the two neighboring VSD signals.

The correlation between the FP and optical signals was assessed by calculating the ratio of IOS or VSD amplitudes (expressed in % of control) for the whole CA1 region and the PS amplitude measured in the CA1 *str. pyramidale*. The correlation between the two optical signals was defined by calculating the ratio of IOS and VSD amplitudes (both expressed in % of control) for the CA1, CA3 and the DG regions.

The spatial pattern of the optical signals during control and drug application was visualized by generating 2D images depicting IOS or VSD amplitudes.

### Statistics

To evaluate drug effects (between the control trials and the drug application from the same region) Mann–Whitney *U* test was used with P < 0.05 considered significant. For regional differences in the optical signal parameters one-way ANOVA was used with P < 0.05 considered significant. Data are reported as means ± SEM. Data processing, analysis, and graphical representations were performed with pClamp9 (Axon Instruments), Origin 9.0 (Origin-Lab) software and custom written scripts in MATLAB 6.5 (MathWorks, Nattick, MA) environment.

## Abbreviations

APV: DL-2-amino-5-phosphonopentanoic acid
CA: Cornu Ammonis
CNQX: 6-cyano-7-nitroquinoxaline-2,3-dione
DAB: 3,3-diaminobenzidine
DG: Dentate gyrus
DHK: Dihyrokainic acid
EAAT2: Excitatory amino-acid transporter type 2
FP: Field potential
Glu: Glutamate
iGluR: Ionotropic glutamate receptor
IOS: Intrinsic optical signal
KCC2: K^+^/Cl^−^ cotransporter type 2
K_ir4.1_: Inwardly rectifying potassium channel type 4.1
K_v4.2_: Voltage-gated potassium channel type 4.2
K_v4.3_: Voltage-gated potassium channel type 4.3
MEA: Multielectrode array
NKCC1: Na^+^/K^+^/Cl^−^ cotransporter type 1
PB: Phosphate buffer
PDA: Photodiode-array
TRPC4: Transient receptor potential cation channel subfamily C member 4
TRPM4: Transient receptor potential cation channel subfamily M member 4
TRPV1: Transient receptor potential cation channel subfamily V member 1
VSD: Voltage-sensitive dye
